# Mirror training to augment cross-education during resistance training: a hypothesis

**DOI:** 10.3389/fnhum.2013.00396

**Published:** 2013-07-24

**Authors:** Glyn Howatson, Tjerk Zult, Jonathan P. Farthing, Inge Zijdewind, Tibor Hortobágyi

**Affiliations:** ^1^Faculty of Health and Life Sciences, Department of Sport, Exercise and Rehabilitation, Northumbria UniversityNewcastle Upon Tyne, UK; ^2^Water Research Group, School of Environmental Sciences and Development, Northwest UniversityPotchefstroom, South Africa; ^3^University Medical Center Groningen, University of GroningenGroningen, Netherlands; ^4^College of Kinesiology, University of SaskatchewanSaskatoon, SK, Canada

**Keywords:** mirror neuron system, rehabilitation, recovery, contralateral adaptations, strength training

## Abstract

Resistance exercise has been shown to be a potent stimulus for neuromuscular adaptations. These adaptations are not confined to the exercising muscle and have been consistently shown to produce increases in strength and neural activity in the contralateral, homologous resting muscle; a phenomenon known as cross-education. This observation has important clinical applications for those with unilateral dysfunction given that cross-education increases strength and attenuates atrophy in immobilized limbs. Previous evidence has shown that these improvements in the transfer of strength are likely to reside in areas of the brain, some of which are common to the mirror neuron system (MNS). Here we examine the evidence for the, as yet, untested hypothesis that cross-education might benefit from observing our own motor action in a mirror during unimanual resistance training, thereby activating the MNS. The hypothesis is based on neuroanatomical evidence suggesting brain areas relating to the MNS are activated when a unilateral motor task is performed with a mirror. This theory is timely because of the growing body of evidence relating to the efficacy of cross-education. Hence, we consider the clinical applications of mirror training as an adjuvant intervention to cross-education in order to engage the MNS, which could further improve strength and reduce atrophy in dysfunctional limbs during rehabilitation.

## Background

A large body of evidence suggests that adaptations in elements of the central nervous system contribute to the responses to resistance training in the trained muscle (Enoka, 1988, 1997; Sale, 1988; Carroll et al., 2001; Aagaard et al., 2002). On a short time scale, adaptive responses in the trained muscle may occur even within one session of motor practice with correlated changes in motor performance and brain activation detected by transcranial magnetic brain stimulation (TMS) and imaging (Muellbacher et al., 2000; Foltys et al., 2003; Cincotta et al., 2004; Perez and Cohen, 2008; Sehm et al., 2010). On a longer time scale, there is now evidence that practice of elementary movements with loads ranging between 20 and 100% of maximum voluntary contraction at a wide range of contraction velocities cause adaptations in the excitability of spinal reflexes, corticospinal pathways, and cortical networks controlling the trained muscle (Carroll et al., 2011). These adaptation have been described independent of age, sex, and training status (Patten et al., 2001; Scaglioni et al., 2002; Kamen and Knight, 2004; Semmler et al., 2004; Kornatz et al., 2005; Ushiyama et al., 2010).

Curiously, neural adaptation to resistance training is not confined to the muscle(s) directly involved in exercise, but becomes expressed in a spatially specific manner in the contralateral homologous muscle in the form of increased voluntary force and neural activation (Hortobagyi, 2005; Carroll et al., 2006; Farthing, 2009). Effortful unilateral motor practice does not result in hypertrophy of the non-exercised contralateral limb muscle in healthy individuals (Hortobagyi et al., 1996a; Farthing et al., 2007), yet fascinatingly, the same exercise can somehow attenuate atrophy and/or strength loss in a disused muscle after short-term immobilization with (Magnus et al., 2013) or without a fracture (Farthing et al., 2009, 2011; Kidgell et al., 2011; Pearce et al., 2012). Another clinical manifestation of this inter-limb interaction is a reduction of muscle damage in a previously non-exercised limb caused by a single bout of eccentric-biased resistance exercise in the contralateral homologous muscle group (Howatson and van Someren, 2007; Starbuck and Eston, 2012). Collectively this adaptive response, commonly referred to as “cross-education,” is the transfer of a motor ability to the contralateral, non-practicing homologous muscle following unilateral practice of a motor task or skill (Zhou, 2000; Lee and Carroll, 2007). The phenomenon suggests that when intact humans practice a unilateral motor task, the active practice on one side of the body can enhance the same motor behavior of the corresponding contralateral homologous muscle even though the muscle is not actively involved in the practice. Reported for the first time over 100 years ago in the psychomotor literature (Scripture et al., 1894), akin to the neural adaptations involved in activating the trained muscle, cross-education has been demonstrated under a variety of conditions along the skill continuum from elementary to complex motor tasks, independent of age, sex, and muscle (Zhou, 2000; Hortobagyi, 2005; Carroll et al., 2006; Lee and Carroll, 2007; Farthing, 2009). Intuitively one would expect that movements with an invariant time and spatial structure that normally make up resistance training would provide insufficient stimuli and produce little or no transfer. However, there is a broad range of evidence showing that repetition of elementary motor skills, as done during resistance training, reliably produces cross-education that is clinically and functionally meaningful (Cannon and Cafarelli, 1987; Hortobagyi et al., 1997; Munn et al., 2004; Farthing et al., 2007, 2011; Kidgell et al., 2011; Pearce et al., 2012; Magnus et al., 2013).

Experimental evidence supporting the strength- and muscle-sparing effects of cross-education during short-term immobilization in healthy individuals with (Magnus et al., 2013) and without (Farthing et al., 2009, 2011; Kidgell et al., 2011; Pearce et al., 2012) a fracture has renewed interest in cross-education as a possible adjuvant therapy in patients displaying unilateral orthopedic and neurological dysfunction. These preliminary findings are promising; however, the duration of dysfunction in many clinical populations can often be much longer than used in previous research and cross-education may not be as efficacious in other clinical groups. Considering that motor transfer occurs to a normally resting limb, it is not unexpected that the magnitude of cross-education was reported to be relatively small, <10% (Munn et al., 2004; Carroll et al., 2006). However, since these publications, evidence from other groups has emerged that lend greater weight to the intervention; these will be discussed in greater detail below. Because of its clinical potential, however, the question is whether there is a mechanism that could augment the magnitude of transfer. Given that sensory feedback during motor practice can increase motor output, one possibility is to activate neurons involved in the transfer that might also be activated by other means, thereby resulting in a synergistic effect on transfer.

In recent investigations (Farthing et al., 2007, 2011; Hortobagyi et al., 2011; Carson and Ruddy, 2012) there is evidence that cross-education following strength training increases brain activation in areas that overlap with areas containing mirror neurons. Mirror neurons are neurons that are activated both during perception and during execution of a motor action (Rizzolatti et al., 1996; Rizzolatti and Craighero, 2004; Iacoboni, 2005). Here we examine the possibility that the mirror neuron system (MNS) could be involved in cross-education and hypothesize that observing the exercising limb in a mirror would augment the magnitude of cross-education. In support of this hypothesis, we provide an overview of the MNS and the evidence surrounding the adaptive response to resistance exercise in cross-education paradigms. More importantly, we present ideas how a mirror might augment these adaptive responses and examine the evidence from brain imaging and stimulation studies that report how observation, imagery and execution of elementary motor tasks modulate brain activity. Finally we review preliminary evidence supporting the role of cross-education in unilateral orthopedic and neurological conditions.

## Overview of the mirror neuron system

The MNS consists of a complex network of neurons distributed over several cortical areas of the brain and provides a neuroanatomical basis for the development of motor learning and skill acquisition, whereby a motor act can be learned and facilitated by observing and imitating the act (Rizzolatti et al., 1999; Rizzolatti and Craighero, 2004; Iacoboni, 2005). The MNS is thought to be important for the development of motor skills and its existence was originally demonstrated in primates (di Pellegrino et al., 1992) and later in humans (Grafton et al., 1996; Rizzolatti et al., 1996). There is a great deal of homology between the primate and human brain, especially in the premotor cortex (Rizzolatti and Craighero, 2004; Small et al., 2012); consequently, primate models provide clues to understanding how the MNS works in primates and also in humans as detailed in comprehensive reviews (Rizzolatti and Craighero, 2004; Rizzolatti and Fabbri-Destro, 2010). The MNS connects neurons responding to visual properties of an observed task with neurons that discharge action potentials when a similar task is executed. In brief, the key points are that the MNS neurons are activated by perceptual input, self-observation of a motor act, observation of a third party's movement, imitation of a motor act, and by movement execution (di Pellegrino et al., 1992; Heyes, 2010; Ray and Heyes, 2011) that are common in the arts and resistance exercise. The MNS comprises of neuronal networks in the visual areas of the parietal, occipital and temporal lobes (Rizzolatti and Craighero, 2004). Areas predominantly activated by motor acts are also core to the MNS in humans (Rizzolatti and Craighero, 2004) and include the inferior parietal gyrus, pre-central gyrus, and inferior frontal gyrus (Rizzolatti et al., 1996; Iacoboni et al., 1999, 2001; Ray and Heyes, 2011).

Observation and execution of motor acts both activate neurons belonging to the MNS. During simple or over-learned tasks, visual information is processed in the superior temporal sulcus (STS) and then sent to the frontoparietal area of the MNS where coding for that specific motor programme occurs. The motor programme is then copied and transferred to the STS where the visual description of the task is compared to the expected sensory consequences of the imitated actions (Iacoboni et al., 1999; Iacoboni, 2005). Prior motor experience is essential (Beudel et al., 2011) and can engage and modulate the MNS, because dancers and musicians, compared with naïve participants, revealed greater mirror activation while observing someone playing an instrument or dancing (Heyes, 2010). Interestingly, when novel tasks are performed there is involvement of additional areas in motor preparation, such as middle frontal gyrus (Rowe et al., 2000), dorsal premotor cortex, superior parietal gyrus and caudal frontal gyrus (Buccino et al., 2004a). Critically, some areas that broadly relate to pars opercularis; namely the ventral premotor cortex, inferior frontal gyrus, inferior parietal lobule are important in adaptive responses and have motor properties that are activated when observing motor actions in a mirror (Molenberghs et al., 2012). To summarize, the frontopariental and STS are involved in the MNS, but additional areas also appear to be implicated when the task is more novel. In the following section, we present evidence suggesting that areas of the brain involved in the MNS might also be associated in cross-education.

## Cross-education following resistance exercise

The transfer of enhanced force-generation to the contralateral homologous “resting” muscle (i.e., cross-education) appears to be influenced by brain areas that are also common with those involved in the MNS. The basis for cross-education with chronic training is that motor areas in both hemispheres become concurrently active during a unilateral muscle contraction, demonstrated by cross-sectional transcranial magnetic brain stimulation (TMS), electroencephalography (EEG) and imaging studies (Kristeva et al., 1979; Cramer et al., 1999; Newton et al., 2002; Zijdewind et al., 2006; Howatson et al., 2011). Many participants show “associated” electromyographic (EMG) activity in the resting muscle during unilateral contractions (Zijdewind and Kernell, 2001; Zijdewind et al., 2006; Sehm et al., 2010). As the performance improves with unilateral resistance training, it is thought that this practice repeatedly excites the relevant brain areas and motor programmes for that task, and become accessible to networks that control the contralateral homologous resting muscle (Carroll et al., 2006; Farthing et al., 2007; Lee and Carroll, 2007; Lee et al., 2009; Hortobagyi et al., 2011).

In one example, participants performed 6 weeks (21–24 sessions) of maximal isometric ulnar deviations with the right arm. Maximal strength increased by 45 and 47% for the trained and untrained arm, respectively. The interpretation was that training modified brain activation and communication between hemispheres, whereby an improved motor plan [from training] provided the untrained brain areas with a reference for preparation and execution for movements (Farthing et al., 2007). Evidence supporting this interpretation comes from accompanying fMRI data showing enlarged regions of activation in the contralateral “trained” left temporal lobe, premotor and visual cortices and “untrained” sensorimotor cortex and primary motor cortex (M1) when participants contracted the homologous muscles of the untrained limb in a magnet (Farthing et al., 2007, 2011). Critically, although the left temporal lobe (especially the STS) and other aforementioned structures appear to be involved in cross-education and the MNS, there is as yet, no direct evidence that the same networks implicated in the MNS are concurrently activated in cross-education. In a separate TMS study, 1000 voluntary isometric contractions of the first dorsal interosseus (at 80% of maximum force, distributed over 20 sessions) increased the excitability of the “untrained” (M1) and decreased interhemispheric inhibition (IHI) by 31% from the trained to the untrained M1. The reduction in IHI correlated with the 28% cross-education (Hortobagyi et al., 2011). Presumably the anterior fibers of the corpus callosum (a structure not implicated in the MNS) mediated such interhemispheric effects between the “trained” and “untrained” frontal motor areas—structures that are involved in the MNS. Indeed, the corpus callosum (specifically the transcallosal pathways) plays a role within the cortical network in promoting a consolidated experience that integrates our perceptions and preparation of our actions (Schulte and Muller-Oehring, 2010). By inference, following training of muscle groups on the right side, when the right sensorimotor cortex and left temporal lobe are implicated in cross-education (reduced IHI) of the muscle groups of the left side, it remains a plausible hypothesis that the MNS is involved since the same brain areas are activated.

## How might the use of a mirror augment the cross-education effect?

Although the exact mechanisms underpinning cross-education following resistance exercise are not fully understood, experimental data in both primates and humans make the expectation tenable that unilateral motor practice (specifically resistance exercise) and cross-education could be enhanced with a mirror. Mirror training involves a superimposed, reflective image of the exercising limb projected on to the non-exercising limb and thereby giving the appearance that the “resting” side is actually active (Matthys et al., 2009; Nojima et al., 2012; Small et al., 2012). Mirror training can increase ipsilateral brain activity (Garry et al., 2005; Matthys et al., 2009), reduce phantom limb pain (Ramachandran et al., 1995; Ramachandran and Rogers-Ramachandran, 1996) enhance recovery of motor function following stroke (Sutbeyaz et al., 2007; Yavuzer et al., 2008) and improve skill acquisition of the non-practiced hand in healthy participants (Hamzei et al., 2012; Lappchen et al., 2012; Nojima et al., 2012). In this section we explore ideas that the overlap between brain areas involved in cross-education and the activation of the MNS might synergistically augment the effect of cross-education with a mirror whilst resistance training.

During a unilateral muscle contraction, there are at least two sources of neural activation that could play a role in the strength- and atrophy sparing effects associated with cross-education. One is the “associated activity” that appears in the resting limb during motor practice with the other limb. The magnitude of the “associated activity” can reach 20% of MVC (Hortobagyi et al., 1997; Zijdewind and Kernell, 2001; Zijdewind et al., 2006) and there is some evidence that resistance exercise at an intensity as low as 10% can improve muscle function (Laidlaw et al., 1999; Duchateau et al., 2012; Kobayashi et al., 2012). Although the source of this “associated activity” is uncertain, it is likely to arise from the hemisphere driving the muscle contraction (Devanne et al., 1997; Zijdewind et al., 2006); repeated and concurrent activation of the motor area controlling the transfer hand could serve a second source for the strength- and muscle-sparing effects seen in cross-education studies (Farthing et al., 2009, 2011; Pearce et al., 2012). As the MNS has overlapping neuroanatomical brain structures with those activated in cross-education, the possibility exists that observing the image of the moving limb in a mirror increases the magnitude of brain activity controlling the resting limb and potentially, the “associated activity” inside the cast.

How such inadvertent brain activity can increase MVC force of the untrained or casted hand is unclear, but one possibility is that the repeated activation of these motor cortical areas changes the threshold of a so far un-recruited subliminal “fringe” of cortical neurons or that the gain of the active neurons increases (Gardiner, 2006). Both would create greater drive during the MVC after the training programme. The use of a mirror during the training could increase the amount of associated activity by engaging the MNS and thus result in a larger training effect. In essence, the use of a mirror creates an action observation effect (discussed in greater detail in the subsequent section) and prime cortical neurons to become more active. Because changes in the maximum slope and threshold of TMS recruitment curves after an intervention reflect the changes in the size of the subliminal fringe (Devanne et al., 1997), it is possible to test whether this mechanism is involved in cross-education (Hortobagyi et al., 2011) and if mirror training amplifies the cross-education effect through this mechanism.

Another possibility for the use of a mirror to augment the transfer effects is an increase in cortical plasticity within and between hemispheres that is evident following unilateral resistance training (Goodwill et al., 2012), thereby further enhancing connectivity following training. In addition, neurons responsible for motor function in the untrained hemisphere that were excitable, but not beyond the point of threshold before training, might be subject to an adaptive response, such that the threshold is either reduced or the corticospinal excitability is increased to the targeted motor neurons and thereby allowing the critical threshold point to be reached, which was not possible before the training. This is conceivable because the magnitude of IHI (Hortobagyi et al., 2011) and short interval intracortical inhibition (SICI) (Hortobagyi et al., 2011; Goodwill et al., 2012) in the untrained cortical hemisphere is attenuated with cross-education effects produced by resistance training. These could be the mediating mechanisms, at least in part, allowing these neurons to reach that “critical” threshold level during and following training, which is akin to theories relating to “motor overflow” (Hoy et al., 2004). Furthermore, imaging studies suggest that previously quiescent areas of the brain become active following unilateral resistance training in the non-trained hemisphere (Farthing et al., 2007). Given the apparent overlap in brain areas of the MNS and those involved in cross-education, the magnitude of response could be further enhanced by specifically activating areas of the MNS by observing the actions of the active limb in a mirror.

The exact mechanisms for these strength increases following cross-education are yet to be elucidated; however, there is the suggestion that the MNS is involved with healthy (Matthys et al., 2009; Nojima et al., 2012) and clinical (Rosen and Lundborg, 2005; Sutbeyaz et al., 2007) populations. Like unilateral resistance training, it seems that mirror training of the right limb (i.e., seeing the mirror image of the exercising right limb) increases the size of the activated brain regions in the ipsilateral (right) M1 (Garry et al., 2005; Nojima et al., 2012), but without causing changes in IHI from the contralateral (left) to the ipsilateral (right) M1 or SICI in the ipsilateral M1 (Nojima et al., 2012). Furthermore, Lappchen et al. (2012) showed increased SICI in the contralateral (left) M1 following right handed skill training with the use of a mirror, which was accompanied by decreased SICI in the ipsilateral (right) M1. This apparent discrepancy in SICI may be due to differences in training duration; Nojima et al. (2012) used a single day as opposed to Lappchen et al. (2012) who conducted 4 days of training, suggesting SICI has a role in the effects of cross-education of skilled motor acts augmented with mirror training. In addition to the ipsilateral (right) M1 (Shinoura et al., 2008; Carson and Ruddy, 2012; Nojima et al., 2012), the right SMA, occipital lobe and cerebellum (Shinoura et al., 2008), and contralateral (left) STS, superior occipital gyrus (Matthys et al., 2009) and M1 (Garry et al., 2005; Shinoura et al., 2008; Tominaga et al., 2011) are involved in mirror training with the right upper extremity. Brain areas that became increasingly active after a short period of mirror training of the right hand were the contralateral (left) inferior parietal lobe and ventral premotor cortex and the ipsilateral (right) dorsal premotor cortex (Hamzei et al., 2012). Enlarged activation areas of the STS, occipital cortex, inferior parietal lobe, premotor areas and the M1 provide links between the MNS, mirror training and performance improvement. It is intuitively appealing to presuppose that combining cross-education with mirror training would stimulate synaptic interactions and thereby strengthen the connections within multiple cortical regions that are known to be involved in the MNS and cross-education, and hence leading to greater levels of cross-education.

The information presented here suggests that adaptations transferred from the practiced “contralateral” brain regions to untrained “ipsilateral” brain regions, activate different networks dependent upon whether they are performed with or without a mirror (Lappchen et al., 2012). Whilst cutaneous and proprioceptive inputs are important and can influence the magnitude of intracortical and interhemipsheric inhibition (Swayne et al., 2006), visual information in the form of a mirror could conceivably facilitate the transfer of strength in a cross-education resistance training model. We suspect from neuroanatomical, electrophysiological, imaging and EEG data that mirror training may be a useful tool to augment the effects of cross-education of strength; however, there are currently no experimental studies that have specifically tested this hypothesis. In the following section we explore the evidence for brain activation during action observation, imagery and the execution of the task.

## Brain activation during movement observation, imagery and task execution

Mirror and imagery training create their effects based on illusionary actions of the resting limb. The inter-limb effects produced by cross-education supplemented with a mirror or done through imagery (Yue and Cole, 1992) would rely on mechanisms also involved in action observation. Many of the areas suggested to have mirror properties, also become active during motor imagery (Grezes and Decety, 2001; Jeannerod, 2001). Jeannerod (2001) described in his “simulation theory” that motor actions have a covert state and both movement observation and motor imagery are covert motor actions. In other words, movement observation and motor imagery are motor actions that have not been executed but that use the same neuronal substrates as the actual performance. Hence, as mentioned briefly in the previous section, movement observation and motor imagery result in subliminal facilitation of neurons that belong to the motor network or, alternatively, the activation of the motor network is actively inhibited before the movement is executed (Jeannerod, 2001; Guillot et al., 2008). Several studies, summarized in reviews, have reported an overlap of brain activation during movement observation and execution (Grezes and Decety, 2001; Jeannerod, 2001; Caspers et al., 2010; Molenberghs et al., 2012) and between motor imagery and execution (Decety, 1996; Grezes and Decety, 2001; Jeannerod, 2001; Munzert et al., 2009). Meta-analyses (Munzert et al., 2009; Caspers et al., 2010; Molenberghs et al., 2012) showed that besides cortical areas that “traditionally” belong to the MNS (ventral premotor area and inferior parietal cortex) action observation and execution involved a bilateral network that comprised premotor, primary somatosensory, inferior parietal, intraparietal and temporo-occipital areas.

The primary motor cortex also shows activity during both motor imagery (Pfurtscheller and Neuper, 1997; Porro et al., 2000; Boeker et al., 2002; Lotze and Halsband, 2006) and motor observation (Molenberghs et al., 2012), albeit less pronounced and its activation is not due to typical mirror neuron activity. The involvement of the corticospinal tract in motor imagery (Kiers et al., 1997; Fadiga et al., 1999; Rossini et al., 1999; Facchini et al., 2002; Fourkas et al., 2006; Roosink and Zijdewind, 2010; Lebon et al., 2012) and movement observation (Fadiga et al., 1995; Rossini et al., 1999; Brighina et al., 2000; Patuzzo et al., 2003; Clark et al., 2004; Roosink and Zijdewind, 2010) has been confirmed with TMS. In single-pulse TMS paradigms, neurons in the motor cortex are activated and modulations in the excitability of the corticospinal tract affects the amplitude of the motor evoked potential (MEP). Many experiments have demonstrated that during movement observation and motor imagery the MEP amplitude is modulated in an effector and task specific manner (Fadiga et al., 1995; Rossini et al., 1999; Facchini et al., 2002; Stinear and Byblow, 2003; Roosink and Zijdewind, 2010; Lebon et al., 2012). Therefore, there is indirect evidence suggesting that the use of a mirror in cross-education interventions could augment the inter-limb transfer through a higher activation of M1 and the corticospinal tract during contraction of the untrained muscles after training.

Although movement observation and imagery activate similar brain areas, the magnitude of activation during observation and imagery is not similar; TMS studies showed differences in the contribution and timing of the corticospinal tract to the effects produced by observation and imagery. Comparisons between TMS evoked responses during movement observation and imagery showed increased activation (Cattaneo and Rizzolatti, 2009; Roosink and Zijdewind, 2010), no difference (Patuzzo et al., 2003; Clark et al., 2004; Leonard and Tremblay, 2007), or decreased activation (Fuerra et al., 2011) during movement observation. Part of these differences could be explained by the use of different tasks; task-specific activation, for instance, increases with task complexity. During execution of a complex motor task, stronger activation foci are seen in the contralateral sensorimotor cortex (Catalan et al., 1998; Kuhtz-Buschbeck et al., 2003), bilateral posterior SMA (Catalan et al., 1998), dorsal premotor area and ipsilateral cerebellum (Catalan et al., 1998; Kuhtz-Buschbeck et al., 2003). Furthermore, single pulse and repetitive TMS studies underline the larger contribution of both the contralateral (Abbruzzese et al., 1996; Gerloff et al., 1998; Roosink and Zijdewind, 2010) and ipsilateral motor cortex (Tinazzi and Zanette, 1998) during complex motor tasks. These data suggest that mirror-aided cross-education studies using complex motor tasks would most likely produce greater inter-limb effects; a hypothesis we are currently exploring using a model that incorporates high intensity resistance training coupled with simple and complex visuomotor skills (i.e., with and without a mirror).

In addition to task complexity, the intensity of muscle contraction also modulates brain activation of the motor areas (Dettmers et al., 1995; van Duinen et al., 2008) and its excitability (Hess et al., 1986; Tinazzi and Zanette, 1998). During observation of tasks that require increased force levels, MEPs produced by TMS increase with the increasing levels of force (Alaerts et al., 2010a,b). The quality of and being an expert in imagery determines which brain areas become active (Guillot et al., 2008) and the intensity of the (subliminal) corticospinal activity (Lebon et al., 2012). Those with high imagery ability showed increased activation of parietal and ventrolateral premotor gebieden; low imagery performers cerebellum, orbito-frontal and posterior cingulated cortices. TMS data also suggests that good, compared with poor imagers, had greater facilitation of the target muscles (Lebon et al., 2012; Williams et al., 2012). Furthermore, skillful vs. less able imagers showed a stronger temporal and spatial modulation of the corticospinal excitability (Lebon et al., 2012). In other words, poor imagers demonstrated a general increase in corticospinal activity that was less focused with respect to timing and the specificity of muscle(s) activation. Furthermore, instructions to the participant are critical; action observation in a passive manner compared to action observation with the intent to imitate, result in similar but weaker fMRI activation (Decety et al., 1997; Grezes et al., 1999; Buccino et al., 2004b; Frey and Gerry, 2006). This observation is also underlined by TMS experiments that showed that the MEP facilitation was larger in the observation to-imitate condition (Roosink and Zijdewind, 2010). Finally, the position of the effector during imagery also affects corticospinal excitability (Vargas et al., 2004; Fourkas et al., 2006). These TMS studies showed a larger facilitation of MEPs when the position of the effector was congruent with the “to-imagine” movement.

Overall these data suggest the action observation elements of mirror-aided cross-education have exciting clinical potential, as long as the intervention conditions are optimized for the population and the individual. Observing one's own movements in a mirror during cross-education therapy is expected to be especially effective if the motor task is challenging, the spatial orientation of the practicing and non-practicing limbs are similar, patients are highly motivated and engaged in the action observation task, and if the act of observation is combined with imagery of the target hand moving (Stefan et al., 2008). In line with these arguments, the subsequent section presents data for the clinical efficacy of cross-education and how this effectiveness could be further increased by the use of a mirror.

## Clinical applications of cross-education and facilitation with mirror-training

A peculiar phenomenon associated with cross-education is that unilateral motor practice does not produce morphological changes in muscles of the non-exercised contralateral limb (Hortobagyi et al., 1996b), yet the same exercise can somehow attenuate skeletal muscle atrophy and/or strength loss in the immobilized limb with (Magnus et al., 2013) or without (Farthing et al., 2009, 2011; Kidgell et al., 2011; Pearce et al., 2012) a fracture. These observations suggest that atrophied and/or injured compared with healthy muscles are more sensitive to neural activation. This section explores firstly, the evidence and application of cross-education in clinical populations and secondly, proposes that the use of mirror therapy might further augment the benefits of strength transfer and attenuate atrophy.

The cross-education effect has long been identified as a potential therapeutic strategy during rehabilitation from unilateral injury or neurological dysfunction. Yet until the recent work on the efficacy of cross-education after wrist fractures (Magnus et al., 2013), ACL surgery (Papandreou et al., 2013) and in post-stroke recovery (Dragert and Zehr, 2013) there was little evidence in patient populations to substantiate this claim. An early attempt by Stromberg (1986, 1988) to apply cross-education during post-operative therapy after various hand and wrist surgeries was not well-controlled and limited by no reporting of pre-surgery status for either limb. Thus, the study received little attention, with misleading reporting of better post-surgery outcomes for the cross-education group. The idea received little further attention until promising evidence emerged from immobilization studies in healthy intact participants, where cross-education was shown to have a strength and muscle sparing effect for the opposite the immobilized arm (Farthing et al., 2009, 2011; Pearce et al., 2012). Unilateral motor practice is not known to produce morphological changes in muscles of the non-exercised contralateral limb, yet strangely, the same exercise can attenuate atrophy in the immobilized limb (Farthing et al., 2009, 2011; Kidgell et al., 2011; Pearce et al., 2012). These observations suggest that atrophied compared with healthy muscles are more sensitive to the effects of neural activation, and the threshold of activity needed to prevent short-term atrophy is much less than is needed to stimulate hypertrophy.

Although the precise mechanisms of the sparing effects remain unclear, Farthing et al. (2011) reported after training of the right limb there was increased activity in contralateral (right) motor cortex and ipsilateral (left) premotor and visual cortices during contractions of the previously immobilized (left) limb; areas that are also associated with the MNS. Pearce et al. (2012) reported unaltered corticospinal excitability and strength maintenance for the immobilized arm of participants who trained the non-immobilized limb, whereas MEP amplitude at various intensities was decreased by ~20% for non-training participants. The data from immobilization models of injury in healthy participants revitalized interest in cross-education as a viable, untested clinical intervention, but lack of patient data remained a key limitation.

Building on the arm immobilization models, there is new evidence that cross-education can benefit the recovery of strength and mobility following wrist fractures. Magnus et al. (2013) implemented unilateral strength training of the non-fractured limb (3 times per week, progressing to 40 maximal efforts per session) within 1-week post-fracture, in addition to standard rehabilitation, in women over the age of 50 who suffered a distal radius fracture. The outcomes for the fractured limb were compared to patients receiving standard rehabilitation alone. The training group had significantly greater fractured limb handgrip strength (~47%) and active range of motion (~25%) at 12 weeks post fracture compared to the control group. Unfortunately, the study was inconclusive for patient's self-rated function scores and there were no measures of muscle or brain activation, or cortical or spinal excitability. However, given there is evidence of increased EMG activity in the non-exercising limb during cross-education studies (Hortobagyi et al., 1997; Zijdewind and Kernell, 2001; Zijdewind et al., 2006), one possibility is that the level of “associated activity” is even greater in the muscle inside the sling or cast during unilateral motor practice than the customary cross-education studies. Notwithstanding, the study is the first to demonstrate the benefit of cross-education in an orthopedic clinical setting involving immobilization. Although the study targets a specific population at higher risk for wrist fractures, the results support the notion that cross-education is probably useful in a broad range of orthopedic injuries that involve unilateral immobilization after a fracture (Farthing et al., 2009, 2011; Kidgell et al., 2011; Pearce et al., 2012).

In addition to the data from wrist fractures, Papandreou et al. (2013) tested cross-education as an adjunct therapy in addition to bilateral strength training to combat quadriceps strength deficit after recovery from anterior cruciate ligament (ACL) reconstructive surgery, in young male soldiers (age 20–25 years) with a recent ACL injury (40 days to 6 months prior). The intervention was eccentric training of the uninjured leg, either 3 or 5 days per week for 8 weeks, using 5 sets of 6 contractions at 80% of 1 RM, completed in addition to a traditional ACL rehabilitation program for both legs (including strength, ROM, balance, and endurance training). Outcomes for the injured leg were compared to a control group who participated in the traditional ACL rehabilitation program only. Although there were no differences between the groups for changes in absolute strength, the cross-education intervention of either 3 or 5 days per week was effective for decreasing the between-leg quadriceps deficit (by ~12% and 17%, respectively) compared to the control group (24%). The studies by Papandreou et al. (2013) and Magnus et al. (2013) support the notion that cross-education is a useful adjunct therapy for a broad range of unilateral orthopedic injury.

Unilateral strength training of the less-affected limb can facilitate bilateral neural plasticity in chronic stroke patients. Dragert and Zehr (2013) demonstrated that intense training of the less-affected dorsiflexor muscles resulted in significant strength gains of 34% for the less-affected limb and 31% for the more-affected untrained limb. The improvements in strength were accompanied by significant gains in dorsiflexor muscle activation, altered reciprocal inhibition, and improved gait speed in a functional walking test. Perhaps most importantly, prior to the intervention four participants were unable to generate functional dorsiflexion in the more-affected limb, but were able to after training of the less-affected limb. This study marks the first evidence that cross-education is a viable strategy to improve bilateral function in a chronic stroke group, particularly when the more-affected limb is initially too weak to train.

Taken together, the wrist fracture, knee surgery, and post-stroke cross-education studies mark important translational advances in the field. As proof-of-principle works, the interventions involved basic isometric strength training or eccentric training of one target muscle group without the use of additional therapeutic strategies such as a mirror. The hypothesis that mirror-facilitated cross-education of strength training would enhance the therapeutic benefit is tenable for both the orthopedic injury and stroke rehabilitation environment. Mirror training has been shown to increase ipsilateral brain activity (Garry et al., 2005; Matthys et al., 2009) enhance skill performance in the resting, non-practiced hand of healthy participants (Hamzei et al., 2012; Lappchen et al., 2012; Nojima et al., 2012), accelerate motor recovery in stroke (Sutbeyaz et al., 2007; Yavuzer et al., 2008), and reduce phantom limb pain (Fadiga et al., 1999; Fourkas et al., 2006). The concept of mirror-assisted cross-education operates on the premise of the illusion that there is more movement in affected or injured limb (by viewing the mirror image of the less-affected or uninjured limb executing intense dynamic strength exercise), and this would further engage common brain areas involved in the MNS (Rosen and Lundborg, 2005; Sutbeyaz et al., 2007; Matthys et al., 2009; Nojima et al., 2012) and further stimulate neural plasticity to augment functional recovery. Unilateral strength (Dragert and Zehr, 2013) and skill training (Ausenda and Carnovali, 2011) of the less-affected limb, and mirror training (Yavuzer et al., 2008; Michielsen et al., 2011) have been shown, independently, to induce bilateral neural plasticity in post stroke rehabilitation. Merging the consequentially enhanced activation of the “ipsilateral” M1 (Garry et al., 2005; Nojima et al., 2012) by mirror training and the known reductions in IHI with chronic unilateral strength training (Hortobagyi et al., 2011) remains a logical next step; albeit firstly in intact participants.

For orthopedic injuries the hypothesis is viable with emerging evidence for the sparing effects in an immobilized fractured limb (Magnus et al., 2013), but there is only sparse clinical case study support for the use of mirror training to re-establish function after cast removal post wrist fracture (Altschuler and Hu, 2008) or after hand surgery (Rosen and Lundborg, 2005). The implementation of mirror training in either context would predictably alter the level of “associated activity” of the opposite affected limb. The associated activity of the fractured limb beneath the cast, during strength training of the non-fractured limb was not examined in the clinical study by Magnus et al. (2013), but the co-activation of more-affected dorsiflexors during training of the less-affected dorsiflexors in stroke patients was reported as 22% post-intervention (Dragert and Zehr, 2013). The associated activity in the resting limb of healthy participants can be as high as 20% of MVC (Hortobagyi et al., 1997, 2011; Zijdewind and Kernell, 2001; Zijdewind et al., 2006), but is commonly around 10% MVC, is position dependent (Post et al., 2009) and diminishes with chronic unilateral training (Hortobagyi et al., 2011). Muscle over-activity and co-activation has been documented post-stroke (Gracies, 2005), which might explain higher levels of post-intervention associated activity reported by Dragert and Zehr (2013). The origin of the associated activity and the clinical relevance of enhancing or reducing this activity during rehabilitation of an injured limb, or a neurologically impaired limb post-stroke are unclear. The hypothesis of augmented cross-education effects by use of a mirror is an exciting premise for future intervention studies in both healthy, and more importantly, clinical populations.

Although the idea surrounding the use of mirror training to engage the MNS and augment cross-education is appealing; the possibility equally exists that the mirror training might not work in further facilitating the response, especially in a patient population. As previously mentioned, patient need a sufficiently challenging task, similar orientation of the practicing and non-practicing limb, be highly motivated and engaged in the observation task, and perhaps combined with imagery of the target hand moving (Stefan et al., 2008). It could be that patient groups are unable to fulfill these requirements because of underlying pain, discomfort, motivation and limb orientation, and hence might compromise the patient's ability to effectively engage in such an action observation task. In addition, it is conceivable that the CNS might prioritize instead of augment activation when using a mirror or that the injury or dysfunction affects sensory pathways involved in mediating the engagement of the MNS.

## General summary

In summary, resistance exercise is a potent stimulus for adaptations in the neuromuscular system. These adaptations are not confined to the exercising muscle and can produce clinically meaningful increases in strength and neural activity in the contralateral, homologous resting muscle. Evidence has shown that these improvements in the transfer of strength are likely to reside in the central nervous system in areas of the brain that are common to the MNS. Given the clinical relevance and importance of this application, we provide a neuroanatomical basis for the, as yet, untested hypothesis that cross-education could be enhanced by augmented sensory feedback using a mirror superimposing the reflected image of the exercising muscle to the non-exercising side and thereby giving the appearance the “resting” side being active. Enhanced cross-education by engaging the MNS with the use of a mirror could improve neuromuscular functionality and the clinical prognosis of many pathologies and is an area worthy of further scientific enquiry. This has the scope to, not only improve strength and reduce atrophy in immobilized limbs during rehabilitation, but also to improve execution of everyday tasks like buttoning shirts, threading needles in other challenged populations.

### Conflict of interest statement

The authors declare that the research was conducted in the absence of any commercial or financial relationships that could be construed as a potential conflict of interest.
